# Monitoring of Assembly Process Using Deep Learning Technology

**DOI:** 10.3390/s20154208

**Published:** 2020-07-29

**Authors:** Chengjun Chen, Chunlin Zhang, Tiannuo Wang, Dongnian Li, Yang Guo, Zhengxu Zhao, Jun Hong

**Affiliations:** 1 School of Mechanical and Automotive Engineering, Qingdao University of Technology, Qingdao 266520, China; zhchl123654@163.com (C.Z.); goodtn1993@163.com (T.W.); dongnianli@qut.edu.cn (D.L.); guoyang@qut.edu.cn (Y.G.); Zhaozhengxu@qut.edu.cn (Z.Z.); 2 Key Lab of Industrial Fluid Energy Conservation and Pollution Control, Ministry of Education, Qingdao University of Technology, Qingdao 266520, China; 3 School of Mechanical Engineering, Xi’an Jiaotong University, Xi’an 711049, China; jhong@mail.xjtu.edu.cn

**Keywords:** monitoring of assembly process, assembly action recognition, segmentation of assembled products, 3D CNN, batch normalization, fully convolutional network

## Abstract

Monitoring the assembly process is a challenge in the manual assembly of mass customization production, in which the operator needs to change the assembly process according to different products. If an assembly error is not immediately detected during the assembly process of a product, it may lead to errors and loss of time and money in the subsequent assembly process, and will affect product quality. To monitor assembly process, this paper explored two methods: recognizing assembly action and recognizing parts from complicated assembled products. In assembly action recognition, an improved three-dimensional convolutional neural network (3D CNN) model with batch normalization is proposed to detect a missing assembly action. In parts recognition, a fully convolutional network (FCN) is employed to segment, recognize different parts from complicated assembled products to check the assembly sequence for missing or misaligned parts. An assembly actions data set and an assembly segmentation data set are created. The experimental results of assembly action recognition show that the 3D CNN model with batch normalization reduces computational complexity, improves training speed and speeds up the convergence of the model, while maintaining accuracy. Experimental results of FCN show that FCN-2S provides a higher pixel recognition accuracy than other FCNs.

## 1. Introduction

During the assembly process of product, if an assembly error is not immediately detected, it may lead to errors and loss of time and money in the subsequent assembly process, and will affect product quality. Using computer vision technology to monitor the assembly process can reduce operating costs, shorten product production cycles, and reduce the rate of defective products. This is especially the case for mass customization production, when assembly lines are often restructured to produce different products. In a changing production environment, assembly quality can often be affected by missed operation steps or by irregular operations of workers. This paper considers the use of computer vision-based monitoring of assembly process, with the aim of quickly and accurately recognizing the assembly action of the workers, and recognizing different parts from complicated assembled products. In this way, assembly efficiency and quality of the products can be improved. Therefore, the research issues of this paper include assembly action recognition and parts recognition from complicated assembled products.

**Figure 1:**
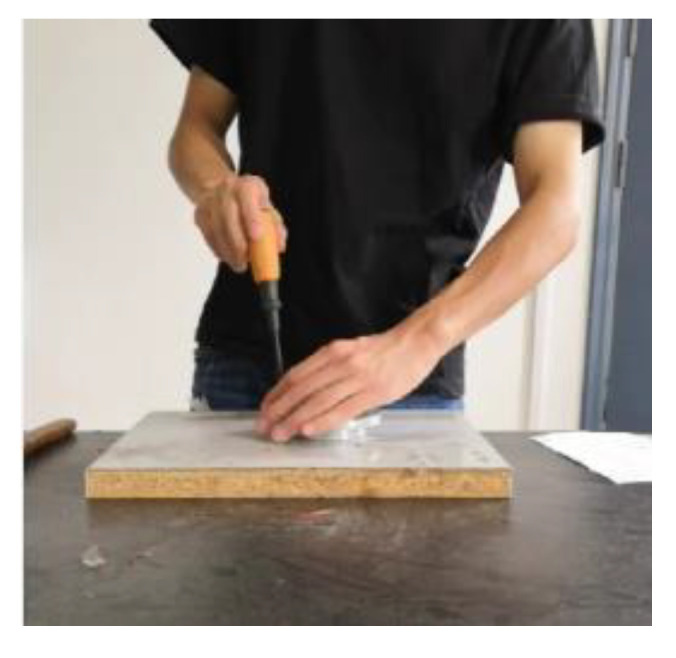
Assembly action.

As shown in Figure 1, the assembly action recognition process falls within the research field of human action recognition. Existing human action recognition methods are mainly divided into two categories according to different feature extraction methods: artificial feature extraction and deep learning. In artificial feature extraction, action features of images or video frames are extracted, and then classified using different kinds of classifiers. For example, Bobick et al. [1] presented a view-based approach to the representation of action. Firstly, motion energy maps of motion features are calculated, and then motion classification is performed by matching with stored action templates. Weinland et al. [2] introduced Motion History Volume (MHV) as action feature for action recognition. This feature can avoid the influence of different human body features on action recognition. Dalal et al. [3] used HOG features to describe human features and classify them with SVM. Chaudhry et al. [4] represented each frame of a video using a Histogram of Oriented Optical Flow (HOOF) and recognize human actions by classifying HOOF time-series. Schuldt et al. [5] constructed video representations in terms of local space-time features and integrate such representations with SVM classification schemes for recognition. Wang et al. [6] introduce the IDT algorithm. The IDT algorithm is currently widely used recognition algorithm based on artificial design features. The algorithm uses dense trajectories and motion boundary descriptors to represent video features for action recognition. Artificial feature extraction-based action recognition methods usually require a complex data preprocessing stage, and recognition accuracy and efficiency can be significantly influenced by feature selection. Deep learning-based methods enable adaptive feature learning with simple data preprocessing and have recently been developed and used in the area of computer vision. Chen et al. [7] studied recognition of repetitive assembly actions to monitor the assembly process of workers and prevent assembly quality problems caused by irregular operation of workers. The YOLOv3 algorithm [8] is applied to judge the assembly tools and recognize the workers′ assembly action. The pose estimation algorithm CPM [9] is employed to recognize the human joint which is subsequently used to judge the operating times of repetitive assembly actions. This paper addresses the problem of assembly action recognition based on deep learning, and proposes a neural network model for assembly action recognition and monitoring.

**Figure 2:**
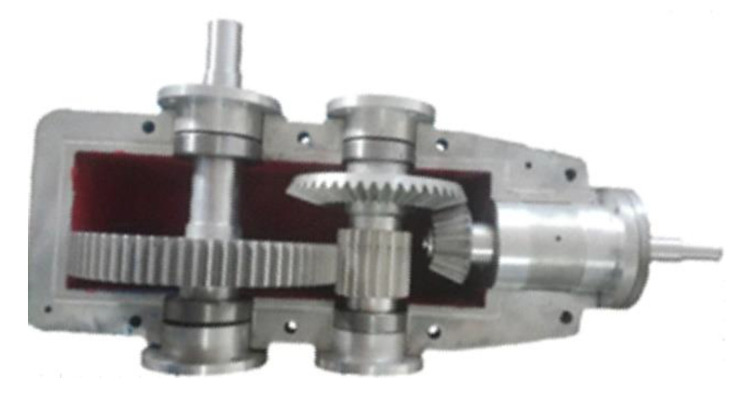
Parts recognition from assembled products.

In mass customization, a wide range of personalized products, with large differences in the assembly process, are produced. Therefore, an assembly monitoring method that detects deviations from the assembly sequence, missing parts, misaligned parts is needed. Therefore, parts recognition is necessary for monitoring assembly process. Computer vision-based assembly monitoring is key to improving the efficiency and the quality of manual assembly. Kim et al. proposed a vision-based system for monitoring the block assembly in ship building. Their system can extract areas of blocks; the extracted blocks are then identified and compared with CAD data in an effort to estimate the assembly progress [10]. Although the abovementioned research realizes the monitoring of the assembly progress, it does not provide part recognition, part positioning and assembly recognition in the overall process. Židek et al. [11] conducted experiments regarding the use of convolutional neural networks (CNN) to achieve a robust identification of standard assembly parts (such as screws, nuts) and features. However, the experiments show that the approach fails to detect parts which are located within a group of overlapping parts and for shiny surfaces which show reflections. As shown in Figure 2, different from the identification of scattered parts before assembling, the assembly of a product usually contains multiple parts which overlap each other. Some parts are only partly exposed due to occlusion, which brings difficulties to detect a whole part from a complex assembly.

The main motivation of this paper is to monitor the assembly process by recognizing assembly action and recognizing parts from complicated assembled products. The main innovations and contributions of the present study are as follows:

(1) We propose a three-dimensional convolutional neural network (3D CNN) model with batch normalization to recognize assembly actions. The proposed 3D CNN model with batch normalization can effectively reduce the number of training parameters and improving the convergence speed.
(2) The fully convolutional networks (FCN) is employed for segmenting different parts from complicated assembled product. After parts segmentation, the recognition of different parts from complicated assembled products is conducted to check the assembly sequence for missing or misaligned parts. As far as we know, we are the first to apply depth image segmentation technology to the application of monitoring of assembly process.

This paper is organized as follows: Section 2 summarizes the state of the art. Section 3 outlines a neural network model for assembly recognition. Section 4 describes the FCN employed for the semantic segmentation of assembled products. Section 5 explained the process of creating data sets. Experiments and analyses that demonstrate the effectiveness and efficiency of our method are provided in Section 6. Section 7 contains our conclusions and future work.

## 2. Related Work

Within the research field of human action recognition based on deep learning, Feichtenhofer et al. [12] proposed a convolutional two-stream network fusion for video action recognition, fusing ConvNet towers both spatially and temporally. A single frame is used as the input to the spatial stream ConvNet, while multi-frame optical flow is the input to the temporal stream. The two streams are fused by a 3D filter that is able to learn correspondences between the highly abstract features of the spatial stream and the temporal stream. This method has a high accuracy, but because of the need to extract the optical flow characteristics of the video in advance, training is slow, and the method is not suitable for long-time video frames.

Wang et al. [13] proposed a temporal segment network (TSN) for video-based action recognition. TSN network is established on the base of two-stream convolutional neural network. In addition to using optical flow graph as input, TSN uses RGB difference and warped optical flow graph as input. Tran et al. [14] proposed a C3D (Convolutional 3D) approach for spatiotemporal feature learning using deep three-dimensional convolutional neural networks (3D CNN) [15]. This method is simple, and easy to train and use. Du et al. [16] proposed a recurrent pose-attention network (RPAN). RPAN is an end-to-end recurrent network. This method uses the postural attention mechanism and can learn some human features by sharing parameters on human joints. Then these features are fed into the aggregation layer to construct the posture correlation representation for temporal motion modeling. Donahue [17] proposed a long-term recursive convolution network (LRCN). In this model, CNN features extracted in time sequence are used as input of LSTM network, which can process time information better. Xu et al. [18] presented a region convolutional 3D network (R-C3D) model. R-C3D firstly extracts features from the network by using the features of C3D network, then obtains the time regions that may contain activities according to C3D features, and finally obtains the actual activity types in the regions according to C3D features and recommended areas. R-C3D can handle any length of video input.

Human action recognition is widely studied, but there are few existing studies relating to assembly action recognition in industry, and there is no public data set for industrial assembly action. Assembly action recognition therefore requires higher recognition accuracy, higher recognition speed, and good adaptability to the working environment, such as changes in light or texture. Most of the traditional artificial feature extraction methods are problematic because of complicated preprocessing, low speed, and poor stability, so are unsuitable for industrial applications.

Common action recognition models based on deep learning include the LSTM-based LRCN model [17], the two-stream convolutional model [10], and the C3D model [14]. The recognition accuracy of these three models on the public data set UCF-101 [19] is similar, but the C3D model is fastest, reaching 313 fps [14], while the two-stream convolutional model is 1.2 fps [12]. This result is due to the fact that the C3D model is relatively simple in its data preprocessing and network structure; the two-stream convolutional model not only needs to process image sequence but also needs to extract optical flow information, slowing it down. Due to the difficulty of the parallelism required for the RNN network, the LRCN model is also slow. Using a 3D CNN for assembly action recognition has the advantages of simple data preprocessing, fast training speed and high recognition accuracy, making it more suitable for industrial field applications.

It is difficult to avoid the influence of changes in illumination intensity on recognition accuracy when simply using the 3D CNN to process RGB video sequences. Under the complex production environment of a factory, it is important to lessen the effect of the environment and improve recognition speed. This paper therefore considers the effects of depth image, binary image and gray image on training speed and accuracy. A 3D CNN model based on the dimensional transformation of single-channel gray video sequences is designed. In addition, the 3D CNN model is improved by introducing the batch normalization layer [20] into the model, which improves the performance of the neural network. Neither single-channel gray images nor three-channel RGB images can affect the understanding of the motion, and gray images can reduce the sensitivity of the model to different illumination conditions. The improved model is shown to effectively reduce the number of training data parameters and to accelerate the convergence and training speeds of the model, while maintaining accuracy.

Semantic segmentation or image segmentation is a computer vision method judging to which object each pixel in image belongs. Shotton et al. [21] mapped the difficult pose estimation problem into a simpler per-pixel classification problem, and a depth comparison feature is presented and used to represent features of each pixel in depth image. Joo et al. [22] proposed a method to detect the hand region in real-time using the feature of depth difference. Long et al. [23] presented fully convolutional networks (FCN) for end-to-end semantic segmentation. FCN has become the cornerstone of deep learning to solve the segmentation problem. Ronneberger et al. [24] presented a U-Net network, which includes an encoding network that extracts context information and a decoding network that accurately locates its symmetric recovery target. The U-Net can achieve end-to-end training using a small amount of data sets. In addition, it has achieved good results in biomedical image segmentation. Zhao et al. [25] proposed a pyramid scene parsing network (PSPNet), which implemented the function of capturing the global context information by fusing the up and down of different regions through the pyramid pool module. The PSPNet network has a good performance in scene analysis tasks. Peng et al. [26] explored the role of large convolution kernels (and effective acceptance domains) in facing simultaneous classification and localization tasks, and proposed a global convolutional network in which the atrous convolution can expand receptive field without reducing the resolution. Chen et al. [27] combined the atrous convolution with the pyramid pool module to propose a new spatial pyramid aggregation algorithm (ASPP). The ASPP can segment the target object at multiple scales.

Li et al. [28] used graph convolution in semantic segmentation, and improved the Laplace algorithm to be suitable for semantic segmentation tasks. Zhong et al. [29] presented a model that uses a novel squeeze and attention module composition (SANet). In order to make full use of the interdependence of spatial channels, the pixel group attention is introduced into the attention convolution channel through the SA module and imposed on the conventional convolution. The outputs of SANet’s four stratification stages are combined. Huang et al. [30] presented the Criss-Cross network (CCNet). The network proposes a crisscross attention module to obtain the context information of all pixels on different paths. Fu et al. [31] presented the stacked deconvolutional network. To fuse context information and restore location information, the network superimposes multiple modular shallow deconvolution networks (called SDN units) one by one. Artacho et al. [32] presented a network based on “waterfall” Atrous space pooling, which not only achieves improved accuracy, but also reduces network parameters and memory footprint. Sharma et al. [33] presented a method which used the DeconvNet as a pre-training network in order to solve the problem of differences between networks in the process of transfer learning.

From the abovementioned research, we can see that the image segmentation technology is mainly used in pose estimation, biomedical image segmentation, scene analysis, face classification and so on. As far as we know, there are few applications of image segmentation in the monitoring of assembled products. In contrast to an identification of scattered parts before assembling, the assembled product usually contains multiple parts which overlap each other. Thus, some parts are only partly visible due to occlusions. For this reason, it is difficult to detect complete parts within a complex assembled product. As shown in Figure 2, compared with a color image, a depth image is less affected by light and texture conditions. Therefore, it is more suitable for the recognition of metal parts. To monitor the assembly process, this paper performs semantic segmentation, which is also known as pixel-wise classification, on the depth image of the assembled product, to determine to which part each pixel belongs. We propose a depth image segmentation method employing FCN [23] to recognize parts from complicated assembled products.

## 3. Three-Dimensional CNN Model with Batch Normalization

The 3D CNN is an extension of the 2D CNN, which adds a time dimension to the base of the 2D CNN. Since there is no need for complex processing of the input sample data, the processing speed of the 3D CNN is faster, making it more suitable for the application of assembly operations. The conventional 3D CNN model [15] consists of input layer, three-dimensional convolutional layer, pooling layer, fully connected layer, and output layer. The input is usually the original RGB video frame or optical flow. Due to the large sample size, the training time is long and the training result is unstable.

In this paper, based on 3D CNN [15] and batch normalization [20], a batch normalization layer is added between the three-dimensional convolutional layer and the activation function on the base of the 3D CNN. The batch normalization layer preprocesses the output of 3D convolutional layer so that its mean value is 0 and its variance is 1, which speeds up the training speed and convergence speed, and improves the generalization of the model. The structure of the improved 3D CNN is shown in Figure 1. Firstly, the continuous video frames are transferred to the three-dimensional convolutional layer, and then the inactivated features obtained from the convolutional layer are transferred to the batch normalization layer. Finally, the features are activated by the ReLu function [34] and transferred to the three-dimensional pooling layer. The features obtained by the last pooling layer are transferred to the softmax function through the fully connected layer for classification and output.

**Figure 3:**
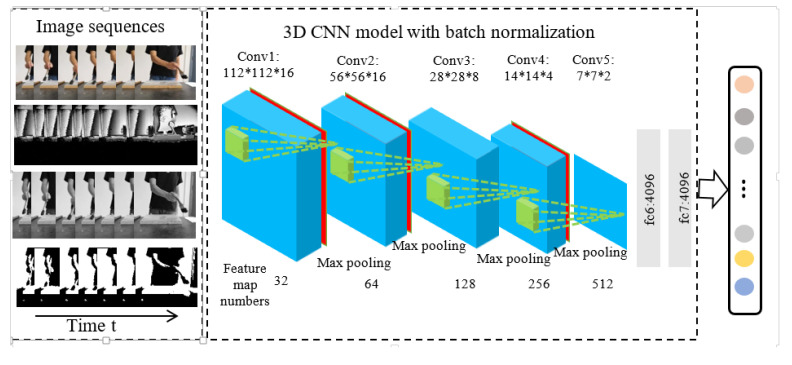
3D CNN model with batch normalization.

The improved 3D CNN model is different from the 3D CNN model [15] by inserting batch normalization layer after Conv1, Conv2 and Conv3. In addition, this paper investigates the effects of gray image, binary image and depth image on training results in addition to the RGB image. Experiments show that the RGB video frame can be transformed into a single-channel gray image by image processing, and its array can be dimensionally transformed to conform to the input requirements of the 3D CNN. Under the 3D CNN model with the batch normalization model proposed in this paper, the training speed can be improved and the convergence time of the network can be reduced while accuracy is guaranteed. The detailed network structure is shown in Figure 3.

The three-dimensional convolutional layer is shown in the blue part of Figure 3. The video frame sequence is used as input of three-dimensional convolutional layer, and three-dimensional convolutional kernel (as shown in the green part of Figure 3) is used to convolute the input video frame. The size of the data inputted into three-dimensional convolutional layer is $a_{1}\times a_{2}\times a_{3}$ (length, height, width), the number of the channel is *c*, and the size of the three-dimensional convolutional kernel is f×f×f, and the convolution kernel dimension is f×f×f×c. If the number of 3D convolutional kernels is *n*, then the output N after the convolutional operation can be expressed as shown in Equation (1).

(1) $$N=\left(a_{1}-f+1\right)\times \left(a_{2}-f+1\right)\times \left(a_{3}-f+1\right)\times n$$

The batch normalization layer is shown in the red part of Figure 3. Like the convolutional layer, the pooling layer and a fully connected layer, batch normalization can also be used as a neural network layer. When each layer of the network is input, a normalization layer is inserted, which is equivalent to preprocessing the data obtained from each convolutional layer, and then entering the next layer of the network to maintain the data between 0 and 1. The transformation and reconstruction methods that are used will not destroy the distribution of the features learned by the convolutional layer. The formula for batch normalization is as shown in Equation (2).

(2) $$\hat{x}=\frac{x^{\left(k\right)}-E\left[x^{\left(k\right)}\right]}{\sqrt{Var[x^{\left(k\right)}}]},$$

where $x^{\left(k\right)}$ represents the neuron parameter, $E[x^{\left(x\right)}]$ represents the mean, and $\mathrm{Var}\left[x^{\left(k\right)}\right]$ represents the variance.

The formula for transforming and reconstructing batch normalized parameters is shown in Equation (3), where $\gamma^{\left(k\right)}$ and $\beta^{\left(k\right)}$ are learnable transformation and reconstruction parameters.

(3) $$y^{\left(k\right)}=\gamma^{\left(k\right)}{\hat{x}}^{\left(k\right)}+\beta^{\left(k\right)}\;$$

Three-dimensional pooling operations are included. Usually, pooling operations include maximum pooling (taking the local maximum) and mean pooling (taking the local mean). The pooling operation can effectively reduce the number of features and reduce the amount of calculation, while also retaining local features. The maximum pooling operation is adopted in this model. The pooling operations were carried out after the first, second and fourth batch-normalized layers and after the third and fifth convolutional layers.

The fully connected layer is shown in the grey part of Figure 3. The main function of the fully connected layer is to act as a bridge between the hidden layer and the output layer (which can flatten the characteristic values of the convolutional layer and the pool layer) and then to transmit the results to the output layer for classification. The dropout processing is often carried out in the fully connected layer, and some nodes are randomly hidden to prevent over-fitting. Another method to prevent over-fitting is L2 regularization, which is shown in Equation (4).

(4) $$J\left(\theta \right)=\frac{1}{2m}\left[\overset{m}{\underset{i=1}{\sum }}{\left(h_{\theta }\left(x_{i}\right)-y_{i}\right)}^{2}+\lambda \left(\overset{n}{\underset{j=1}{\sum }}\theta_{j}^{2}\right)\right]$$

where $\sum_{i=1}^{m}{\left(h_{\theta }\left(x_{i}\right)-y_{i}\right)}^{2}$ is the loss function, *θ* is the parameters of the CNN model, $\lambda \left(\sum_{j=1}^{n}\theta_{j}^{2}\right)$ is the regular term, and *λ* is regularization coefficient.

The output layer is classified by the softmax function.

## 4. FCN for Semantic Segmentation of Assembled Product

**Figure 4:**
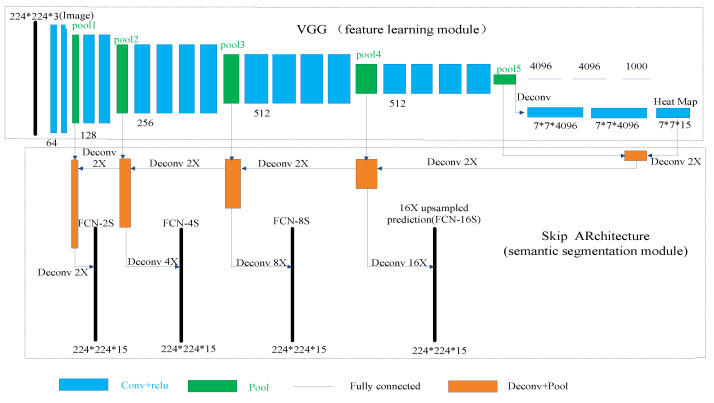
The FCN structure.

As can be seen in Figure 4, we use the FCN for the semantic segmentation of a depth image of an assembled product. The FCN can be divided into two stages: the feature learning stage and the semantic segmentation stage. In the feature learning stage, the VGG classification nets [35] were reinterpreted as fully convolutional nets. We further use the transfer learning [36] approach to retrain the parameters of the convolution layers of the VGG with the depth images and pixel labeled images of the assembled products. The semantic segmentation stage is composed of a skip architecture, which combines coarse, high layer information with fine, low-layer information. The combined semantic information is up-sampled to the dimension of the input image using the deconvolution layer. Therefore, a label prediction for each pixel is generated while preserving the spatial resolution of the input image. Using the predictions of all pixels, a semantic segmentation of the depth image of an assembled product is obtained.

The lower the selected layer is, the more refined the obtained semantics are. Therefore, on the basis of the FCN-8S nets, lower layers were used for the up-sampling to generate more refined semantic segmentations. We further defined FCN-4S and FCN-2S nets as well as FCN-16S nets to compare the effects of semantic segmentation. All above mentioned nets are shown in Figure 4.

## 5. Creating Data Sets

### 5.1. Creating the Data Set for Assembly Action Recognition

**Figure 5:**
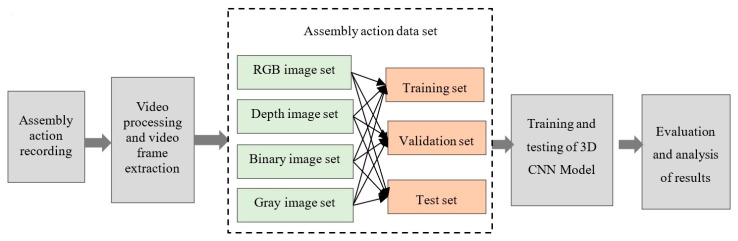
Flowchart for creating the data set for assembly action recognition.

Training a 3D CNN requires a data set with enough sample, followed by the creation and training of the model. The network structure is then continuously adjusted through analysis of the results to obtain the appropriate 3D CNN model with batch normalization. The process is shown in Figure 5.

The creation of an assembly action data set is required before the neural network can be trained. There is currently no assembly action data set, so this research includes the creation of such a set. The RGB video and the depth video were simultaneously recorded by a Kinect depth camera, and the video frames were separately extracted from the two videos. Assembly action is different from common human actions (e.g., running, jumping, squatting, etc.). It is mainly upper body movement, which is usually repeated, using appropriate assembly tools. Many assembly actions are similar but the tools used may be different, so tool information is also useful for recognizing assembly action.

**Figure 6:**
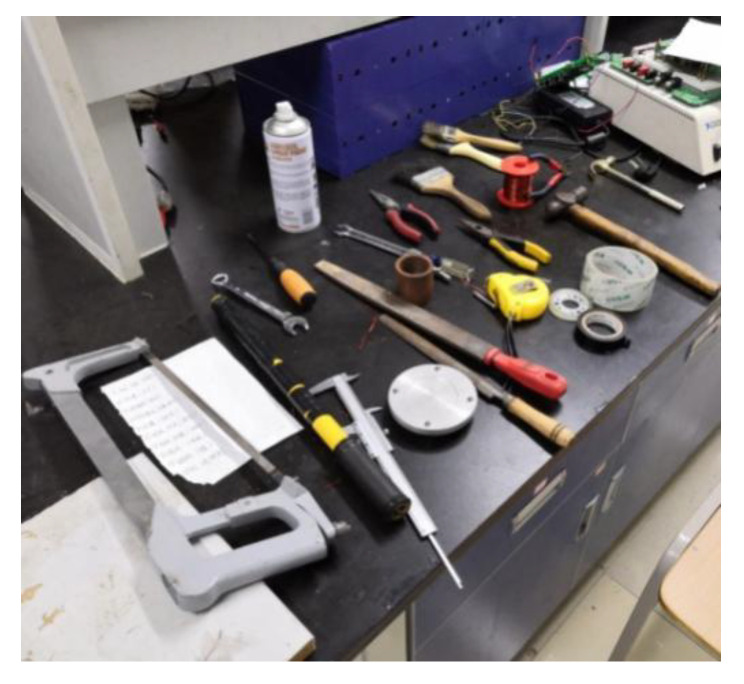
Assembly tools.

The data set of assembly actions created for this research includes nine kinds of common assembly actions (screw twisting, nut twisting, hammering, tape wrapping, spray painting, brushing, clamping, sawing and filing) each of which is operated by 12 people (the ‘participants’). To ensure data set diversity and to enhance the generalization characteristics of the assembly actions, two or three tools are provided to finish each assembly action, chosen by the participants. When recording the video, the participant performs the corresponding assembly action with respect to his own understanding of the action. The assembly tools are shown in Figure 6.

**Figure 7:**
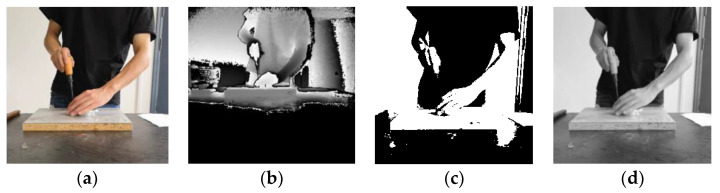
Comparison of the four image types of data set. (**a**) RGB image; (**b**) Depth image; (**c**) Binary image; (**d**) Gray image.

The video for each type action for each participant was edited and divided into three or four video data samples, which were each associated with one of the nine assembly action classification labels. Each action category contained 36 segments of video data samples, each of which ranged between 3 and 6 s in duration. Both deep video and RGB video adopted the same processing method. The RGB images were converted into gray images and binary images, respectively. Accordingly, four types of data set were obtained: RGB video sequence, depth image video sequence, gray image video sequence, and binary image video sequence. Figure 7 shows the four different types of data sets images corresponding to the same assembly action.

The RGB image is a color image consisting of three primary colors of red, green and blue. Each picture contains three channels of information, and the values of each channel are 0–255. The RGB image is rich in content, but it has three color channels and is sensitive to changes in light intensity.

The depth image with depth information is obtained using the Kinect depth sensor; the position information contained in each pixel reflects the distance from the sensor.

The binary graph is obtained by binarizing the RGB image, using only the values of 0 and 1 for each pixel. It results in loss of image information to varying degrees, and it is difficult to distinguish what the experimenter is doing with a single-frame image.

The gray image is a single channel image, which no longer contains color information, and the range of values for each pixel is 0–255. The gray image can reduce the amount of data while ensuring the integrity of the image information.

### 5.2. Creating the Data Set for Image Segmentation of Assembled Products

**Figure 8:**
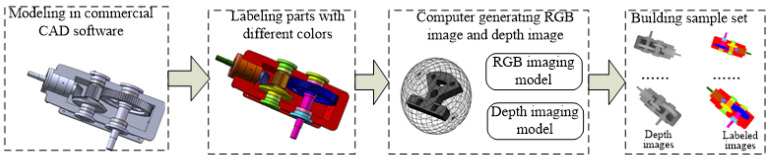
Flowchart of creating image segmentation data set.

As shown in Figure 8, we design a flowchart to create the sample set for training FCN model to recognize parts from complicated assembled products. The process for computer generating depth images and labelling RGB images is as follows:

(1) Commercial CAD software such as SolidWorks is selected to build the CAD model of the product and the CAD model of the product is saved in obj format.
(2) Mutigen Creator modeling software is used to load the assembly model in obj format. Each part in the assembly model is labeled with one unique color. Therefore, different parts correspond to different RGB values. The assembly models for different assembly stages are saved in OpenFlight format.
(3) The Open Scene Graph (OSG) 3D rendering engine is used to design an assembly labeling software, which can load and render the assembly model in OpenFlight format, and establish the depth camera imaging model and RGB camera imaging model. By changing the viewpoint orientation of the depth camera imaging model and RGB camera imaging model, the depth images and RGB images of product in different assembly stages and different perspectives can be synthesized by a computer.

Using the above process, the data set for image segmentation of assembled products can be synthesized by computer without using the physical assembly. Therefore, it is suitable for training FCN model to recognize parts from personalized products, which is usually not produced before monitoring.

## 6. Experiments and Results Analysis

The system used in this experiment is Ubuntu 16.04 (64 bits), the graphics card is NVIDIA QuadroM4000 and the CPU is Intel E5-2630 V4 @ 2.20 GHz × 20, and 64G RAM. Experiments for both recognizing assembly action and recognizing parts from complicated assembled products are conducted.

### 6.1. Assembly Action Recognition Experiments and Results Analysis

#### 6.1.1. Assembly Action Recognition Experiments

The Adam optimization algorithm [37] is used. The basic network structure in the experiment was first determined based on the training results of the RGB data set of assembly action. Subsequently, the batch normalization layer was introduced and tested on different data set images to adjust the network structure. Ultimately, the four data sets were compared and evaluated. The sample size of all data sets is identical, with the same comprising 16-frame sequence images in the sub-folder under each action classification. That is, the input is 16 × 112 × 112 × 3 or 16 × 112 × 112 × 1, where 3 and 1 are the number of channels. Table 1 shows the settings of the parameters and functions using in the CNN model. Three quarters of each data set were randomly selected as the training set, with 20% of them being the validation set. The rest quarter of each data set is the test set.

**Figure 9:**
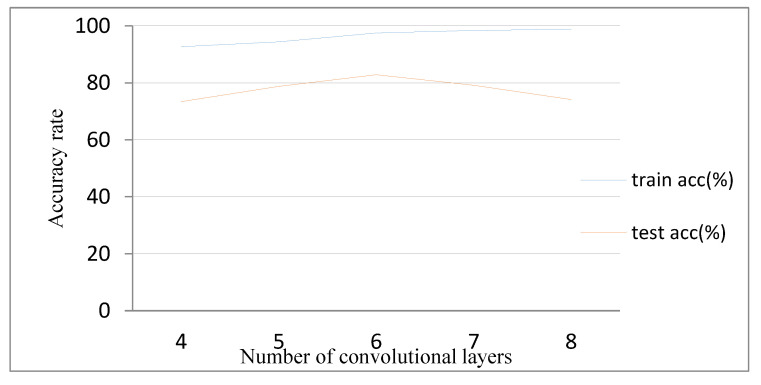
Comparison of network depth.

First, the 3D CNN model is built based on the RGB data set. The model structure adopts the structure of the C3D model [14], comprising only a stack of a three-dimensional convolutional layer and a three-dimensional pooling layer. Figure 9 shows the comparison between the training results of different convolutional layers. When the number of three-dimensional convolutional layers is four and five, the accuracy of the test set deviates greatly from that of the training set, and the training result is in an under-fitting state. When the number of three-dimensional convolutional layers is seven and eight, the deviation between the test set accuracy and the training set accuracy is gradually increased, and the phenomenon of over-fitting appears. When the depth of convolutional layer is six, the 3D CNN model achieves better results. In the absence of any preprocessing of the data set, the accuracy of the test set is 82.85%.

Next, the structure of the 3D CNN model is finally determined by introducing the batch normalization layer, adjusting the model, and testing and optimizing on different types of data set. As shown in Figure 3, the 3D CNN model with batch normalization consists of five three-dimensional convolutional layers, five three-dimensional pooling layers, three batch normalization layers and two fully connected layers.

The dimensions of the single channel data sets are then transformed to conform to the input requirements for the 3D CNN. For example, the size of the gray image is 112 × 112, a two-dimensional matrix which cannot be used as the input to the 3D CNN. The dimension of the gray image is thus transformed into 112 × 112 × 1.

Finally, the four types of data set of assembly action are used as input to the improved 3D CNN model. The training results are compared and analyzed with respect to the four criteria of stability, training time, convergence speed and accuracy.

#### 6.1.2. Analysis of Experimental Results

##### Comparison of Stability and Convergence Speed

**Figure 10:**
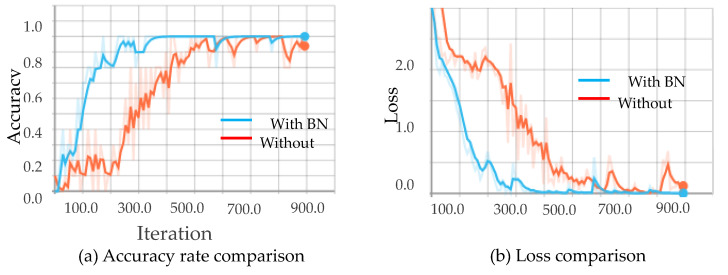
Comparison curves for the RGB image.

**Figure 11:**
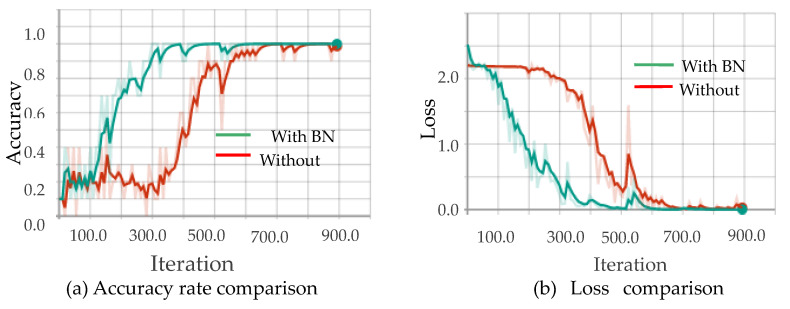
Comparison curves for the binary image.

**Figure 12:**
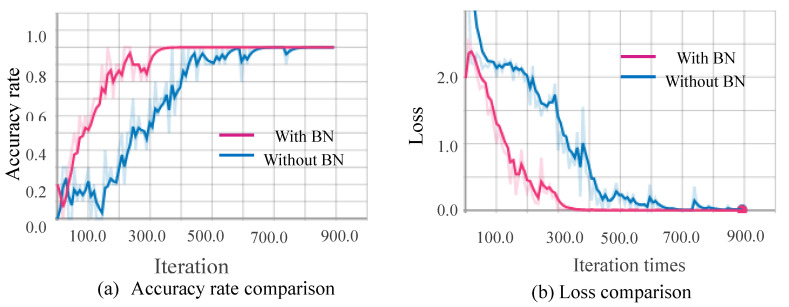
Comparison curves for the gray image.

**Figure 13:**
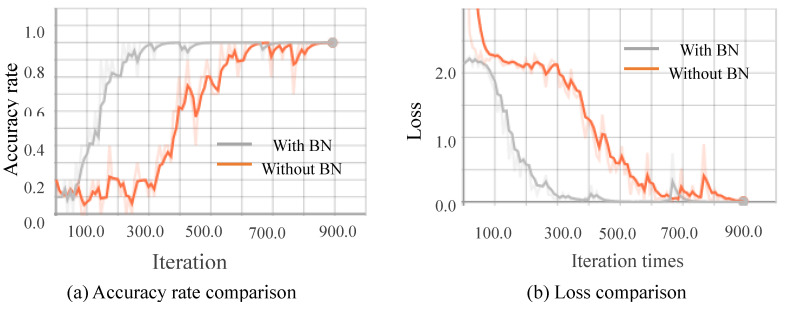
Comparison curves for the depth image.

Figure 10, Figure 11, Figure 12 and Figure 13 show the results obtained from training using the four data sets of assembly action. The (a) part of the figures show accuracy comparison, in which the ordinates are the accuracy and the abscissa are the number of training samples. The (b) show loss comparison, in which the abscissa is the loss values and the abscissa is the training steps or Iteration times. The “With BN” and “Without BN” curves show the difference between the results with and without the batch normalization layer, respectively.

**Figure 14:**
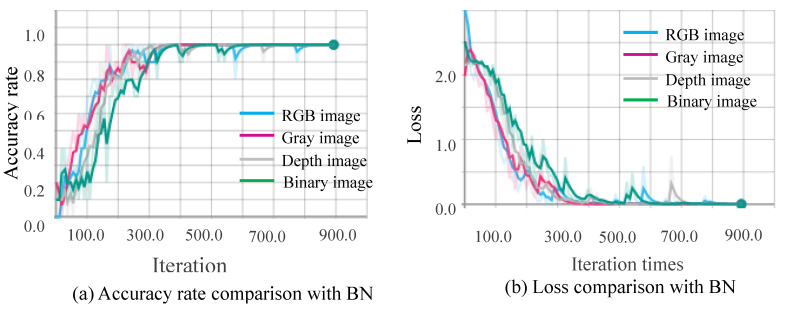
Comparison of training for four data sets.

From Figure 10, Figure 11, Figure 12 and Figure 13, we can see that the introduction of the batch normalization layer improves the convergence speed for the RGB video sequence, the binary image video sequence, the gray image video sequence, and the depth image video sequence, and the improved 3D CNN model provides better stability on the training set. Figure 14 shows a comparison of the training results of the batch normalization layer model on the four data sets.

The convergence speeds of training using the video sequences of the binary image and the depth image are slightly slower than that of the RGB image and the gray image, and the effect of the binary image is the worst.

##### Comparison of Accuracy and Training Time

The training results of the common 3D CNN model and the improved 3D CNN model on the four different types of data sets are compared and tested with the test set. The introduction of the batch normalization into 3D CNN model [15] should improve the initial learning rate and improve the training speed. For the sake of fairness, the initial learning rates of the common 3D CNN and improved 3D CNN are set to be the same to avoid the impact of the learning rate on the training speed. Table 2 shows the comparison of accuracy and training time for different test sets. The accuracy and of each data sets is the average of 10 tests.

In comparing the four data sets, the training times for the binary image and the depth image are slower, confirming the result shown in Figure 14; that is, that the convergence speeds of the binary image and the depth image are slower than that of the other two types. The training speed can be improved significantly by transforming the RGB image into gray image through image processing. In addition, the introduction of the batch normalization layer does not directly improve the training speed, but since the batch normalization layer can improve the convergence speed, the training time can be reduced by decreasing the number of training iterations.

The accuracy of the RGB video sequences is highest owing to the abundant picture information, followed by the gray video sequence, but there is little difference between these two video sequences. In gray image data set, the identifying accuracy of screw twisting, nut twisting, hammering, tape wrapping, spraying, brushing, clamping, sawing, and filing is 75%, 80%, 78%, 80%, 87.5%, 78%, 88%, 75% and 90%, respectively. The average speed for recognition of an action is about 18.8 fps.

**Figure 15:**
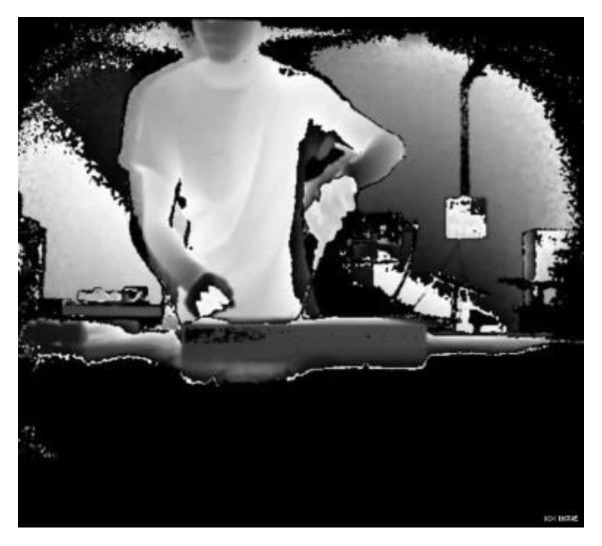
Screw twisting.

**Figure 16:**
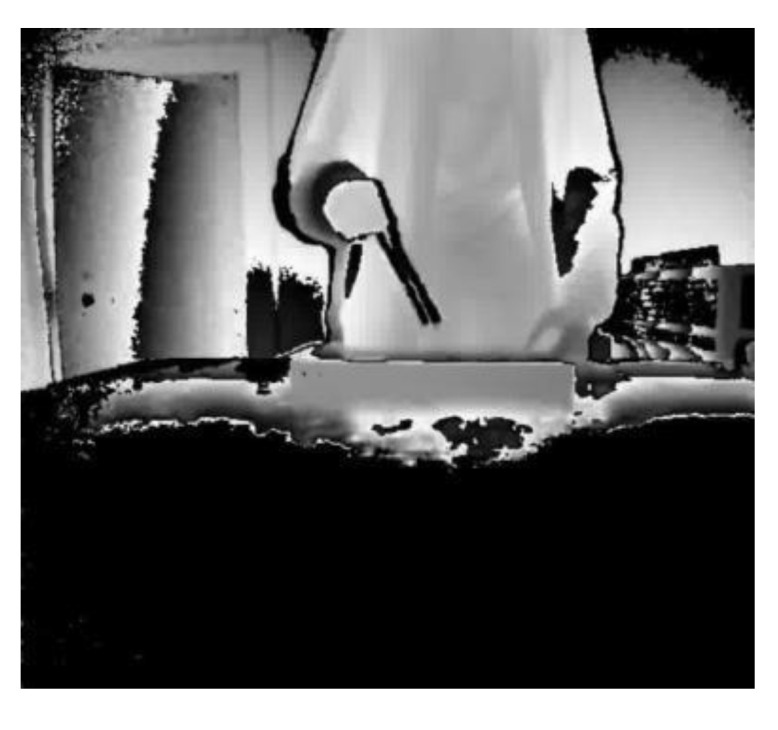
Brushing.

However, both the depth image and the binary image will lose image information to varying degrees, resulting in low test results. This is particularly the case for the depth image, when there may be serious misjudgments. When the depth image is acquired, the true depth value can be recorded. The depth value (700–4000 mm) when represented by a gray scale of 0–255 will bring a depth error of about 15 mm. Figure 15 and Figure 16 show the depth video frames of the assembly actions of screw twisting and brushing, respectively. It is difficult to see from the picture which tool is in hand and what the participant is doing.

Analysis of the experimental results has shown that the 3D CNN with fusion batch normalization can effectively reduce the number of training parameters and improve the convergence speed. The single-channel grayscale image video sequence can preserve image content well, and training speed is improved while ensuring training precision.

### 6.2. Parts Recognition Experiments and Results Analysis

As shown in Figure 2, a gear reducer consisting of 14 parts and a worm gear reducer consisting of seven parts are used as the assembled product. The gear reducer and the worm gear reducer were modeled using 3D modeling software. Each part of the assembled product is marked with a unique color. 3D models of different assembly phases were rendered using the Open Scene Graph (OSG) rendering engine. Using a depth buffer technology, depth images with different viewpoints for different assembly phase can be generated. For each product, in total of 180 computer-generated depth images were obtained; 120 computer-generated depth images contribute to the training set and 60 computer-generated depth images contribute to the validation set. In addition, 10 depth images of a physical assembled object are used as the test set. In training the FCN, the training set and the validation set were increased to 405 and 135, respectively, by the data augmentation method. A transfer learning strategy was employed to initialize the FCN.

In order to evaluate the performance of the proposed methods, the accuracy of the pixel classification (as shown in Equation (5)) is used as one of the evaluation criteria. The pixel classification accuracy PA is defined as follows:

(5) $$PA=\frac{P_{Y}}{P_{N}},$$

where *P_Y_* is the number of correctly predicted pixels, *P_N_* is the total number of pixels.

Table 3 shows the FCN network parameter configuration and the number of parameters. FCN-2S has only a slight increase in FCN-2S compared to FCN-8s in terms of the number of network parameters.

**Figure 17:**
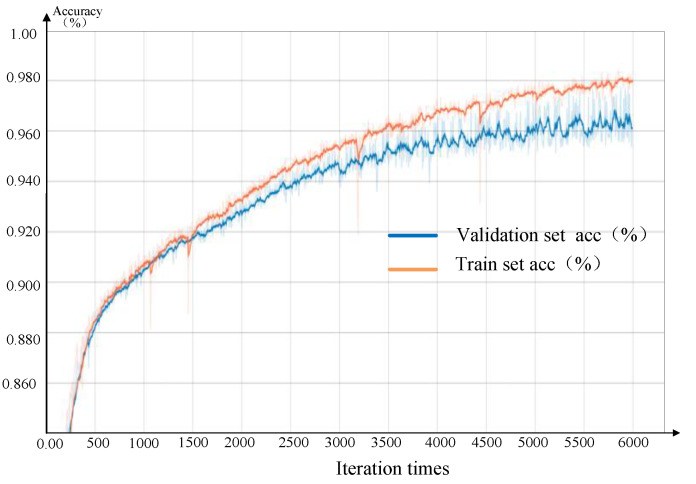
Training accuracy of FCN.

Figure 17 shows the pixel classification accuracy of the FCN-8S network and the number of iterations. With an increase in the iteration time, the pixel classification accuracy of both the training set and the validation set improve. After 6000 iterations, the final pixel classification accuracy of the validation set is as high as 96.1%. The online training took 5.81 h. The above results show that the proposed method based on FCN for assembly monitoring achieves a good performance.

Table 4 shows the comparison of the pixel classification accuracy for the test set and the validation set. From the comparison of the pixel classification accuracy and the network training time between FCNs with different output structures, it can be seen that the FCN-2S has the highest accuracy regarding pixel classification on the test set. The average run time of pixel classification for one depth image is about 0.173 s.

**Figure 18:**
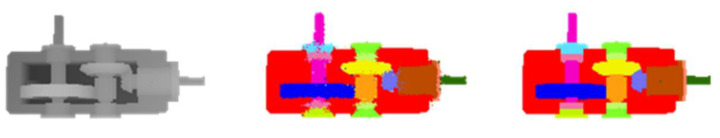
Depth image segmentation of gear reducer.

**Figure 19:**
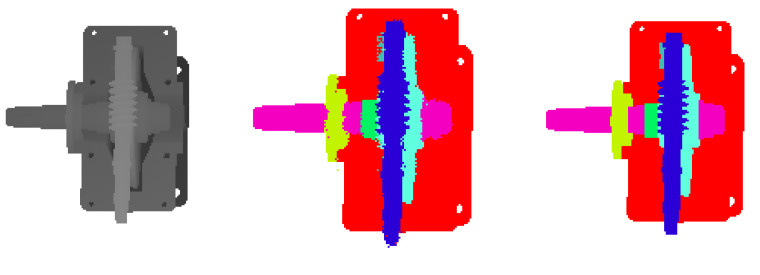
Depth image segmentation of worm gear reducer.

As shown in Table 4, the experimental results of the gear reducer show that the FCN-4S and FCN-2S are 1.62% and 2.7% higher than FCN-8S in pixel accuracy. The pixel accuracy of FCN-16S is 2.26% lower than that of FCN-8S. For feature learning, the FCN uses the invariant method of spatial transformation, which also limits the spatial accuracy of the object. The lower-level convolutional layer has accurate location information. In the lower-level convolutional layer, the FCN network can learn accurate location information, thereby improving network performance. The FCN-2S network has reached 98.80% pixel accuracy, and the test pixel accuracy has reached 94.95%. The experimental results of the worm gear reducer also show that FCN-2S has achieved the best results, with an accuracy rate of 99.53%, which is 1.7% higher than FCN-8S. The test pixel accuracy has reached 96.52%. In summary, the FCN-2S network has achieved the best results in the data set for image segmentation of mechanical assembly. Figure 18 and Figure 19 show the segmentation of depth images. The left figure shows the depth image inputting into the FCN-2S model. The middle figure shows the output of FCN-2S. The right figure is the ground truth of the left depth image.

## 7. Conclusions and Future Work

In this paper, a 3D CNN model with batch normalization is proposed. An assembly actions data set including a gray, binary, depth and RGB image is created. The 3D CNN model with batch normalization is tested on the four types of data set images. The experimental results show that the improved 3D CNN model with batch normalization can effectively reduce the number of training parameters, reduce the computational complexity, improve the training speed and convergence speed, while maintaining accuracy. These results are a significant contribution to the research on recognition and monitoring of assembly action and assembly quality in the mass customization production. The FCN-based semantic segmentation method is employed to segment parts from complicated assembled products. The experimental results demonstrate that the FC-2S network provides the highest pixel classification accuracy and the fastest run time. Both the 3D CNN model with batch normalization and the FCN-based semantic segmentation method can thus serve the purpose of online monitoring the assembly process of mass customization production. Future work may include research on pose estimation of each part in product, and judging whether each part is placed to its right place. It is also possible to combine the four types of images in the same decision system, even combine assembly action monitoring and semantic segmentation to improve the results in both tasks.

## Figures and Tables

**Table 1:** 3D CNN parameter configuration.

Method	Crop Size	Loss Function	Optimizer	Learning Rate	Batch Size	Decay Rate	Decay Steps
3D CNN	112 × 112	Cross_entropy	Adam	0.0001	10	0.5	2

**Table 2:** Comparison results for the four data sets.

Data Set Type	RBG Image	Binary Image	Gray Image	Depth Image
Accuracy (without BN)	82.85%	79.78%	80.86%	70%
Accuracy (with BN)	83.70%	79.88%	81.89%	68.75%
Training Time (without BN)	50 m 37 s	54 m 34 s	46 m 45 s	55 m 9 s
Training Time (with BN)	51 m 10 s	54 m 35 s	48 m 3 s	55 m 50 s

**Table 3:** Network parameter configuration and number of parameters.

Method	Image Size	Loss Function	Optimizer	Learning Rate	Batch Size	The Number of Parameters
FCN-16S	224 * 224	Cross_entropy	Adam	0.00001	1	145259614
FCN-8S	224 * 224	Cross_entropy	Adam	0.00001	1	139558238
FCN-4S	224 * 224	Cross_entropy	Adam	0.00001	1	140139998
FCN-2S	224 * 224	Cross_entropy	Adam	0.00001	1	140163614

**Table 4:** Comparison of pixel classification accuracy.

Method	Data Set	Pixel Classification Accuracy (PA) (Gear Reducer)	Pixel Classification Accuracy (PA) (Worm Gear Reducer)
FCN-16S	validation set	93.84%	97.64%
FCN-8S		96.10%	97.83%
FCN-4S		97.72%	98.59%
FCN-2S		98.80%	99.53%
FCN-2S	test set	94.95%	96.52%
